# Intracranial pressure and venous sinus pressure gradients: what happens 3 months after stenting?

**DOI:** 10.1186/2045-8118-12-S1-O65

**Published:** 2015-09-18

**Authors:** Hasan Asif, Claudia Craven, Syed N Shah, Simon D Thompson, Aswin Chari, Samir A Matloob, Neekhil A Patel, Edward W Dyson, Patricia Haylock-Vize, Andrew R Stevens, Huan Wee Chan, Jinendra Ekanayake, Tarek Mostafa, Ahmed K Toma, Laurence D Watkins

**Affiliations:** 1 Victor Horsley Department of Neurosurgery, National Hospital for Neurology & Neurosurgery, Queen Square, London, UK

## Introduction

Benign Intracranial hypertension (BIH) is commonly associated with venous sinus stenosis. Increasingly, this is treated endovascularly with stent insertion. However, this treatment modality is still controversial. Clinical improvement post stent insertion has been described. Little is known about long-term control of intracranial pressure (ICP). In our unit, catheter cerebral venogram with pressure measurements is routinely performed 3 months post stent insertion in BIH patients. We aim to quantify the degree of venous pressure changes in stenosis patients treated with sinus stenting and how the changes correlate with radiographic improvements.

## Methods

Single Centre case series. Clinical, angiographic and intracranial pressure data before and 3 months after stent placement were reviewed. All venograms were done under local anesthetic in supine position.

## Results

Between 2011 and 2015, 33 patients underwent post stent insertion cerebral venogram as a routine follow up. Mean pre-stent superior sagittal sinus pressure was 28.8 ± 2.0mmHg (mean±SEM). Mean 3-months post-stenting superior sagittal sinus pressure was 10.5 ± 0.7mmHg (p<0.0001). Pre-stenting pressure gradients across stenosis were reduced from 18.3 ± 11.9mmHg to 6.3 ± 4.5mmHg 3-months post-stenting (p<0.0001). 25 of the 33 patients showed radiological stent patency and stenosis obliteration. Five developed new focal narrowing distal and three proximal to the stent.

## Conclusions

This study provides objective evidence of the effectiveness of venous sinus stent insertion in reducing intracranial pressure 3 months post procedure in the majority of patients with intracranial hypertension and focal venous sinus stenosis. Angiographic evidence of patent sinuses correlated with reduction in pressure gradients.

